# A Proposed Mechanism for Development of CTE Following Concussive Events: Head Impact, Water Hammer Injury, Neurofilament Release, and Autoimmune Processes

**DOI:** 10.3390/brainsci7120164

**Published:** 2017-12-19

**Authors:** Steven Kornguth, Neal Rutledge, Gabe Perlaza, James Bray, Allen Hardin

**Affiliations:** 1 Department of Kinesiology and Health Education, The University of Texas at Austin, Austin, TX 78712, USA; 2 Department of Neurology Dell Medical School, The University of Texas at Austin, Austin, TX 78712, USA; 3 Research Imaging Center, Austin Radiological Association, Austin, TX 78705, USA; nrutledge@austin.rr.com; 4 Department of Intercollegiate Athletics, The University of Texas, Austin, TX 78712, USA; gabe.perlaza@athletics.utexas.edu (G.P.); james.bray@athletics.utexas.edu (J.B.); allen.hardin@athletics.utexas.edu (A.H.); 5 Department of Population Health, University of Texas, Austin, TX 78712, USA

**Keywords:** chronic traumatic encephalopathy, brain structure and function, concussion, water hammer effect, neurofilament release from brain, autoimmune disease

## Abstract

During the past decade, there has been an increasing interest in early diagnosis and treatment of traumatic brain injuries (TBI) that lead to chronic traumatic encephalopathy (CTE). The subjects involved range from soldiers exposed to concussive injuries from improvised explosive devices (IEDs) to a significant number of athletes involved in repetitive high force impacts. Although the forces from IEDs are much greater by a magnitude than those from contact sports, the higher frequency associated with contact sports allows for more controlled assessment of the mechanism of action. In our study, we report findings in university-level women soccer athletes followed over a period of four and a half years from accession to graduation. Parameters investigated included T1-, T2-, and susceptibility-weighted magnetic resonance images (SWI), IMPACT (Immediate Post-Concussion Assessment and Cognitive Testing), and C3 Logix behavioral and physiological assessment measures. The MRI Studies show several significant findings: first, a marked increase in the width of sulci in the frontal to occipital cortices; second, an appearance of subtle hemorrhagic changes at the base of the sulci; third was a sustained reduction in total brain volume in several soccer players at a developmental time when brain growth is generally seen. Although all of the athletes successfully completed their college degree and none exhibited long term clinical deficits at the time of graduation, the changes documented by MRI represent a clue to the pathological mechanism following an injury paradigm. The authors propose that our findings and those of prior publications support a mechanism of injury in CTE caused by an autoimmune process associated with the release of neural proteins from nerve cells at the base of the sulcus from a water hammer injury effect. As evidence accumulates to support this hypothesis, there are pharmacological treatment strategies that may be able to mitigate the development of long-term disability from TBI.

## 1. Introduction

The study reported here was designed to prospectively assess the mechanisms by which emerging traumatic brain injury (TBI) and subsequent development of clinical chronic traumatic encephalopathy (CTE) in athletes participating in contact sports are affected by the repeated rapid acceleration and deceleration of the brain [1,2,3,4,5,6,7,8,9,10,11]. Clinical manifestations of TBI occur in approximately one third of collegiate and professional football and soccer players during their lifetime, although a much larger percentage of players experience major body impacts [12]. The authors suggest that clinical neural dysfunction is a result both of the susceptibility of the athlete/soldier to neural injury and of the forces impacting the head. In a study, over 3000 high school athletes who played football in 1957 were examined in the sixth decade of life and the authors reported that there was no association in these athletes between playing football and increased incidence of dementia [13]. 

There is no current algorithm that determines the probable emergence of CTE in an athlete/soldier years after impact. A 2002 report [2] from the Institute of Medicine (IOM) identified soccer as a sport in which young athletes experience significant brain injury, and a 2011 report [1] from the IOM discussed cognitive rehabilitation therapy for TBI in athletes. Recent publications have documented the effects of injuries that may contribute to CTE experienced by football players, boxers, and women soccer players on cognitive capability and function [9,10,14,15,16,17,18,19,20]. From the publications cited, it is apparent that women soccer players experience mild to moderate brain injury to an equal or greater extent than other women athletes [4].

Blast impacts experienced by soldiers exposed to improvised explosive devices (IEDs) in theater [9] and repetitive head impacts experienced by athletes in high contact sports exhibit similar changes in brain structure and subsequent cognitive capability years after the traumatic experiences. The forces impacting the blast-exposed soldiers from IEDs are approximately a magnitude higher than that of the athletes even while the athlete experiences high-velocity head impacts repetitively [8,9]. For example, the force experienced by a football running back (200 lb) being hit at full acceleration (5 m per s) by an opposing lineman (200 lb) approximates 800–1000 Newtons [21]. 

Earlier studies presented by Nizamutdinov and Shapiro [20] review the molecular, cellular, and systemic responses to traumatic head and body events. These authors report a release of macrophages from spleen into blood and their subsequent penetration of the central nervous system CNS compartment at regions of disrupted blood–brain barrier (BBB). There are increases in levels of inflammatory response modifiers consistent with an autoimmune role in the development of CTE [9,11,20,22]. Studies by Wang et al. discussed the potential role of autoimmune processes involving glial fibrillary acid protein in emergent TBI [23]. Bargerstock et al. and Marchi et al. discuss the potential role of repeated blood–brain barrier permeability in the initiation of TBI [24,25]. 

Shahim [14], Bernick [16], Oliver [19], and Rubenstein [10] demonstrated the increased permeability of the BBB following concussive events; Shahim [15] also demonstrated that biomarkers (light neurofilament protein, tau protein, etc.) are released into blood and that these biomarkers may be correlated with injury sufficient to cause long-term neural dysfunction after several years. The Kornguth laboratory demonstrated [26,27,28,29] that patients with small cell cancer of the lung (SCCL) who exhibited visual paraneoplastic syndrome were observed to have elevated levels of antibodies to the light neurofilament (l NF) protein, and several of these patients had antibodies to the medium and heavy neurofilament proteins. These antibodies to the NF proteins reacted with the small cell cancer as well as the large retinal ganglion cells affected by the visual paraneoplasia. The anti-neurofilament antibodies resulted in the selective immunoablation of large retinal ganglion cells following injection of these antibodies into cat vitreous [29,30]. These observations on the SCCL population are supportive of the observation by Shahim [14,15] that the l NF proteins seen in the mild TBI (mTBI) pass from the neuronal compartment into the cerebrospinal fluid (CSF) over a prolonged time period. The continued release may serve as an antigenic stimulus to generate anti-NF antibodies in blood serum and, subsequently, to the destruction of neuronal populations in the CNS. The etiology is hypothesized in this paper to be an autoimmune process that can be anticipated to have periods of exacerbation and remission, even as the long-term consequences lead to neural dysfunction and cognitive loss. 

To more clearly explore the chain of events that may lead to CTE, our research team recruited women soccer players at the university level at the time of their accession to college and examined them using magnetic resonance imaging MRI, cognitive metrics, neurological and physical examination, and academic performance on a yearly basis for five years. The results of our study are reported here with a view toward evaluating the potential role of an evolving autoimmune process that may lead to CTE. The hypothesis has clinical implications because there is a significant literature base describing treatment protocols for the management of autoimmune disease processes, as discussed below. 

## 2. Methods

Women soccer players at the university level were asked to volunteer to participate in the magnetic resonance (MR) imaging study at the time of their acquisition into the university. All the athletes were informed of the goals of the research and potential risks as indicated in the Institutional Review Board approved protocols. The consent to participate was obtained. No financial or other compensations were provided to the participants. The MR imaging studies included T1-, T2-, and susceptibility-weighted imaging (SWI), and these studies were performed when the athletes entered the University, at yearly intervals as well as within 96 h following a concussion. The clinical evaluations (neural, cardiovascular) and the IMPACT (Immediate Post-Concussion Assessment and Cognitive Testing) and C3 Logix assessments were performed on all athletes. All athletes underwent a complete pre-participation physical examination on arrival, and were followed up annually with questionnaires regarding their health status. When the athletes suffered a concussion, a physical exam focused on that diagnosis was repeated several times until the player returned to normal status.

The MRI scanner used was the Siemens Skyra 3T, Erlangen, Germany. The T1-weighted images reveal myelinated rich areas that appear bright, while in T2-weighted images the gray matter appears bright. The susceptibility-weighted images (SWI) provide indication of blood vessels in the brain and of deposits of iron from hemoglobin that has effused from the vascular bed into the perivascular region of the brain parenchyma. 

A total of 21 women athletes (19 were soccer players at the University level) were studied. Ten of these soccer athletes were scanned on a yearly basis over a multiyear period and T1, T2, and SW images were acquired. With this approach, it was possible to have each individual serve as their own control with regard to structural changes observed from imaging and to IMPACT and C3 Logix assessments. 

The brain volume metrics were calculated completely unsupervised using single timepoint workflows in Freesurfer 5.3.0 (Martinos Center for Biomedical Imaging, Massachussetts General Hospital MGH, Harvard, MA, USA) as validated by Reuter, Schmansky et al. [31]. Data were parsed and analyzed using in-house written code in MATLAB R2015b. Benchmark data were obtained from the MyConnectome project [32] and were acquired over the course of approximately one year on the same scanner during the same time period as the TBI-related data presented here. The single human subject of that study was a healthy 45-year-old male in excellent physical condition with no history of diagnosed TBI before or during the study.

## 3. Results

Among the athletes who were scanned multiple times, there was one (Athlete 15) who had experienced a substantial concussive event as determined from clinical evaluation by the team physician during the five year period of study. Two additional players (Athletes 19, 29) experienced concussive events but they were not imaged over a four year period of competition. No clinical evidence of substantial concussive events was reported for the remaining athletes during their competitive activity at the University level even though multiple subclinical events may have occurred. Athlete 16 was the only player in the study who was reported to have experienced a concussive event prior to her university activities. All of the athletes in this study participated in soccer activities for 10 to 14 years prior to entering the university program.

### 3.1. Observations from MR Imaging

**Figure 1:**
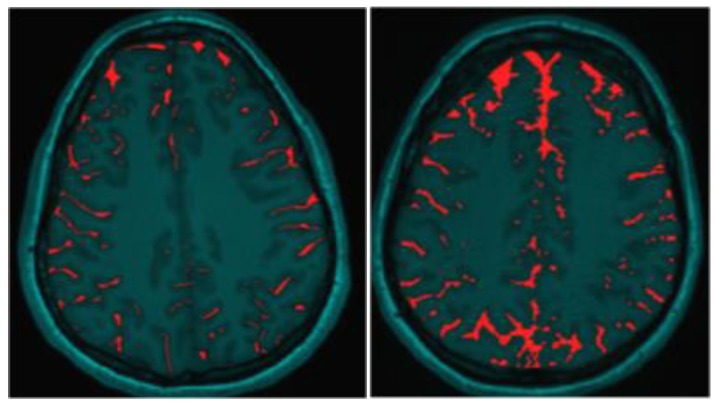
Illustration of increased Sulcal Volume in the brain of Women Soccer players before (**left** image) and after (**right** image) concussive events. The red coloring indicates the Sulcal volume.

**Figure 2:**
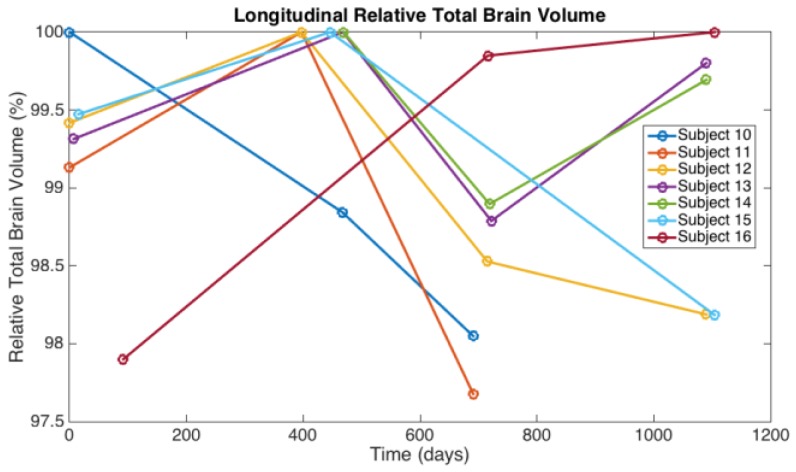
The longitudinal relative total brain volume of seven women soccer athletes who were assessed over a four year period.

**Figure 3:**
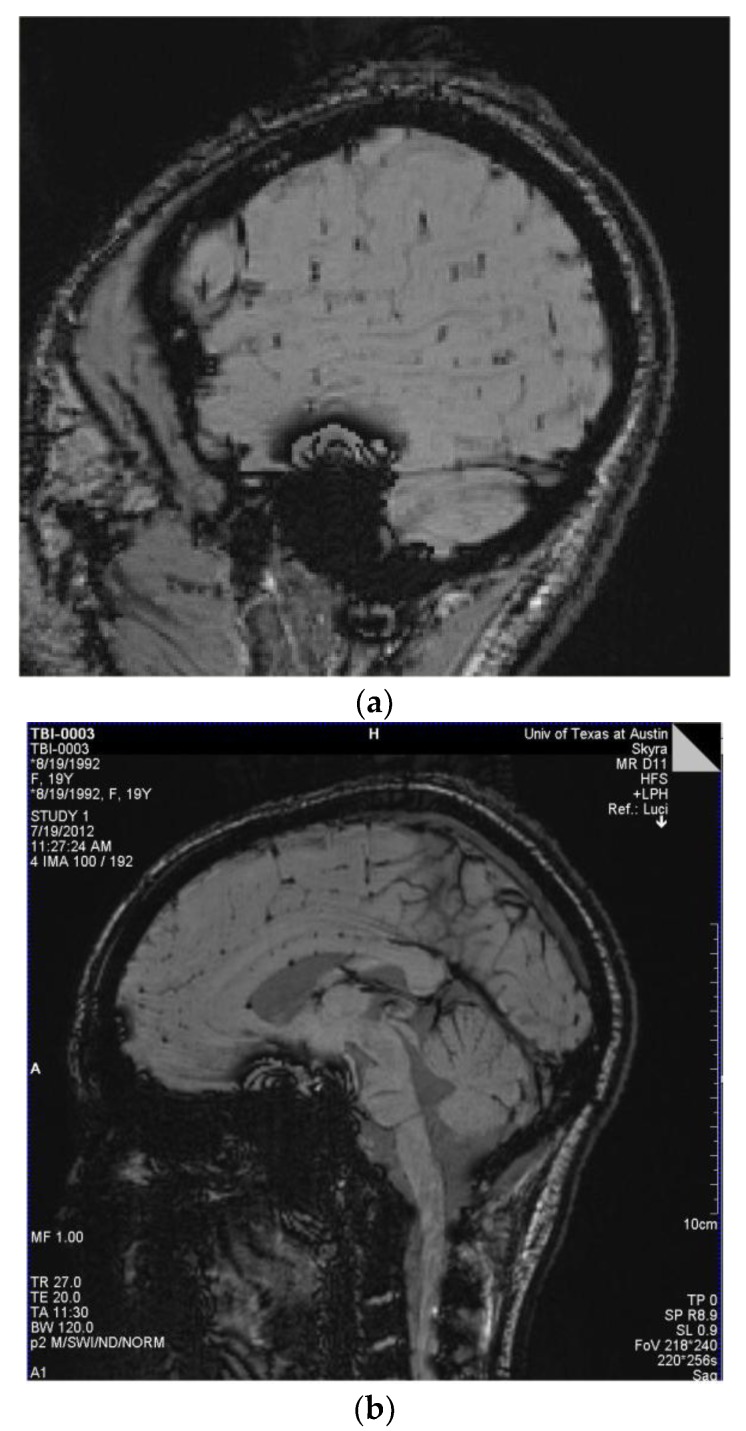
(**a**) Players 29 and 16: Susceptibility-weighted imaging (SWI) demonstrating multiple small areas of signal loss in the base of the sulci. Paramagnetic compounds including deoxyhemoglobin, ferritin, and hemosiderin from the hemorrhages distort the magnetic field resulting in the signal loss. These images are consistent with a primary site of vascular injury and bleed into the brain parenchyma at the base of the sulci; (**b**) SWI Image of Player 12 demonstrating multiple small areas of signal loss in the base of the sulci. Paramagnetic compounds including deoxyhemoglobin, ferritin and hemosiderin from the hemorrhages distort the magnetic field resulting in the signal loss.

**Figure 4:**
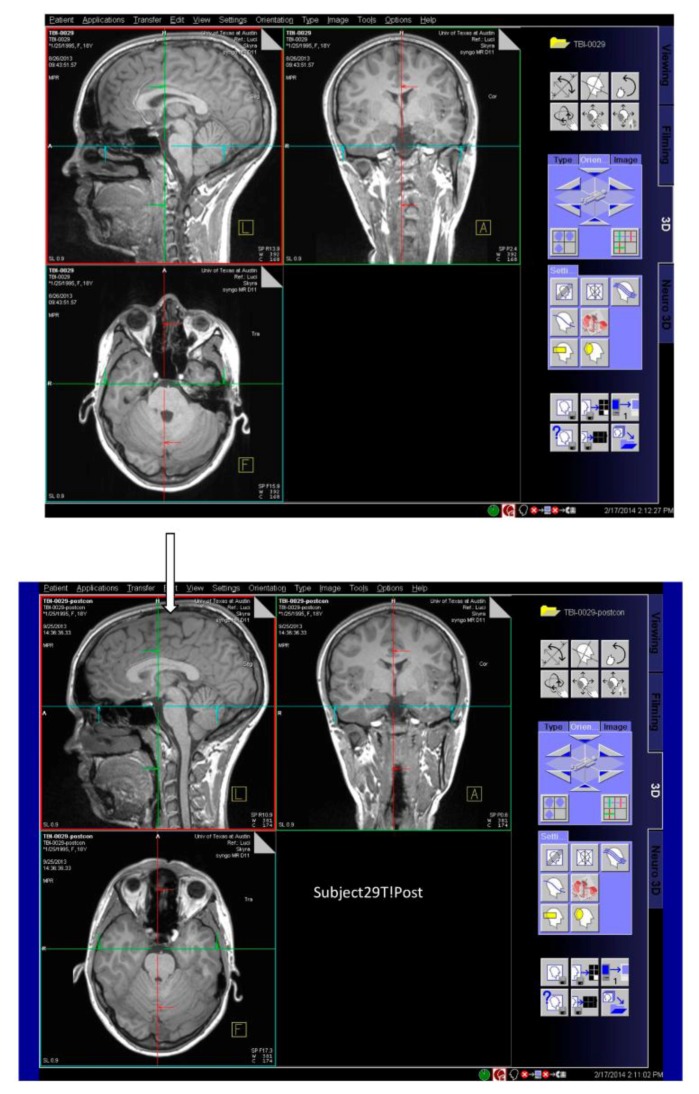
Images in three planes of a woman athlete before concussive event (**upper** image); three planes of images of the same athlete several days after concussive event (**lower** image). The widened sulci under the arrow in the region of the pre- to post-central sulci after concussion are visible compared with the preconcussion image in (**upper** image).

The most readily observed changes were increases in the width of the sulci in many of the players, as illustrated in Figure 1. The most profound changes appeared in the athletes who had experienced concussive events but the sulci were also widened in the soccer players who did not have reported concussions. Because we observed increases in width of the sulci in players, the changes in brain volume of each individual athlete were calculated to quantify the changes as described in the Methods section. The changes in the calculated longitudinal relative total brain volume of seven athletes over the years is shown in Figure 2. In addition to the widening of the sulci in the frontal, parietal, and occipital regions of the brain, we observed low-intensity punctate regions in the white matter interfaces on T2- and susceptibility-weighted images associated with the base of the sulci (Figure 3). Four of the players imaged over four years exhibited reductions in brain volume (Athletes 10, 11, 12, 15) while the remaining athletes exhibited no change in relative brain volume over the multiyear period. Only one of the players with reduced brain volume, Athlete 15, sustained a concussive event while the other three did not have reported concussions. She had a concussion during her first year at the University. An additional athlete (Player 29) was evaluated using MRI (Figure 4, pre- and post-concussion) and the IMPACT tool following the concussion and then again four days later. The MRI revealed increases in the sulcal volume in the region of the pre- to post-central sulci following the concussive event. Athlete 29 was not imaged for four consecutive years and so was not included in Figure 2. From these observations, it appears that overt concussive events are not required for there to be a reduction in brain volume. The reduction of two percent in the longitudinal relative brain volume of the four athletes is unusual when compared with the metadata analysis reported in Hedman et al. [33]. The brain volume in normal women between the ages of 18 and 25 generally increases two to three percent or remains the same during this developmental stage; this is the age range of athletes in our study.

From the susceptibility-weighted (SW) imaging, it appeared that several of the subjects had potential vascular injuries, including the individual who experienced a concussion. The vascular injuries occurred in athletes who exhibited no overt concussive events, indicating that such injuries can occur absent the clinical signs of concussion.

### 3.2. Observations from IMPACT and C3 Logix Assessments

The data from the IMPACT and C3 Logix batteries are shown in Table 1. The numbers for Subject 15 indicate baseline scores, the scores one day following head concussions (asterisk), and the scores four days later (two asterisks). The numbers for the subjects shown in this table are for the same individuals as the numbers shown in Figure 2. 

It is noteworthy that the individual who experienced a concussion also had a reduced brain volume during the fourth year of competition and had a concentration score of 2/5. Her cognitive efficacy as measured by IMPACT showed a transient reduction from 0.2 to 0.08 but within four days the cognitive efficacy score returned to normal levels. The delayed memory C3 Logix score was excellent for this player. Two other athletes (Subjects 10 and 14) had scores of near 0.2 in the cognitive efficacy IMPACT measure. Neither of these athletes exhibited a reduction in brain volume.

## 4. Discussion

The most prominent observation in this study was the marked increase in the width of the sulci of soccer players and the determination that the increased width was most evident in the athletes who experienced concussions. The reduction in brain volume of some soccer players was at an age when meta-analyses indicate that normally brain growth occurs rather than reduction in brain volume [33]. A reduction in brain volume of a similar magnitude following mild traumatic brain injury was reported by Jarrett and colleagues [34]. Brain volume changes can vary as shown by Poldrack et al. [32], but our changes show a persistent and increasing atrophy. The significance of the finding of reduced volume of brain of the women athletes over a four year period remains to be determined because of the longitudinal study of brain volume in a single adult who was in his fifth decade of life [32]. Our study reveals no clear association between overt concussive events that occurred during collegiate competition and reductions in brain volume. Changes in brain volume similar to those reported here were observed over the one year daily study of that individual; however, the consistent decrease in volume of the brain of the women athletes over the four year time span during an age when the brain is generally increasing in volume suggests that this finding may be significant. 

**Figure 5:**
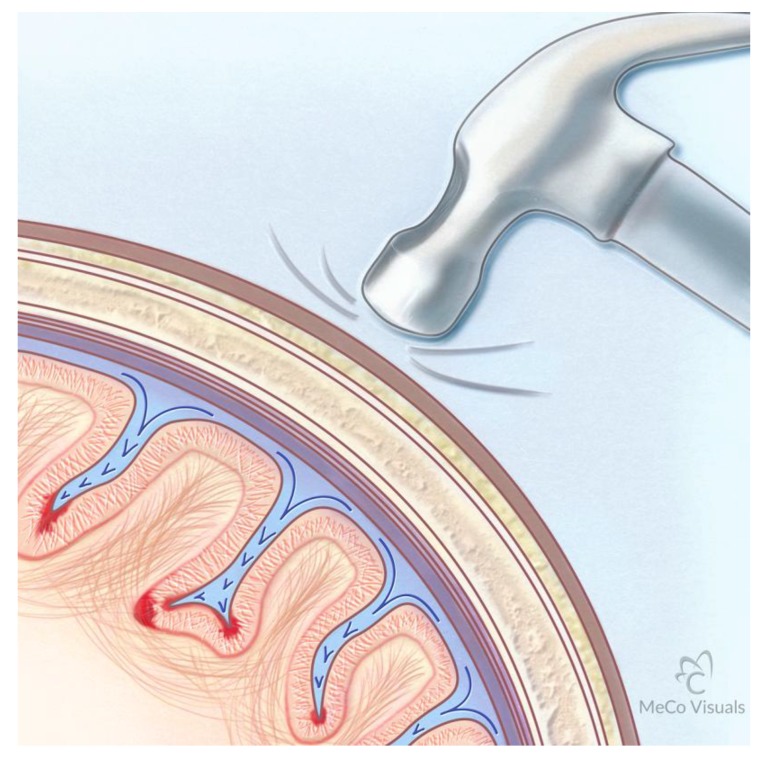
Water Hammer Illustration of the mechanism whereby traumatic impact to the skull results in transmission of force to the cerebrospinal fluid (CSF). As the elastic brain impacts the non-compressible calvarium, the non-compressible CSF is driven into the sulci. The base of the sulcus receives the major force of the CSF impulse. The alignment of the axons in the gray matter at the base of the sulcus is oriented parallel to the vector force while the U fiber bundles at the base are oriented perpendicular to the vector. Differing rigidity features of the gray and white matter result in shearing at that interface. The areas of red intensity at the base of the sulci represent sites of major force dissipation. The intense linear red at the interface between the gray matter and the U fiber bundles represents areas of bleeds from vascular injury.

The widened sulci suggest that the brain is deformed during repetitive impact of heads as a result of rapid acceleration/deceleration of an elastic brain interacting with non-compressible spinal fluid and a non-compressible cranial vault during heading of the ball and collisions between players. The widening of the sulci, and the presence of low-intensity punctate regions in the white matter interfaces with gray seen on SW and T2 images of our soccer study, is indicative of a “water hammer” causation during high-velocity head impacts [35]. During the impact, the deformable brain parenchyma is driven against a non-compressible cranial vault and the non-compressible CSF is then driven into the sulci, as illustrated in Figure 5. The region of brain exposed to the highest force is at the base of the sulcus where the “water hammer” force must dissipate if brain integrity is retained. The regions at the base of the sulcus are primary sites of vascular injury and hemorrhage into the brain parenchyma. Post mortem evidence obtained from brains of athletes who had TBI is consistent with the base of the sulci being most involved pathologically [11]. Neuronal damage consisting of axonal bulbs and swellings is most commonly located in the deep gyri at the interface between the gray and white matter [11]. The SWI reported here shows small focal hemorrhage where small vessels and U fibers are impacted. 

The vulnerability of the interface between gray and white matter of the brain to injury from water hammer effects results from both the differing mechanical properties of the gray and white matter as well as the orientation of major dendritic and axonal processes at the base of the gyri. The white matter is, on average, 39% stiffer (average modulus 1.895 kPa) than gray matter (1.389 kPa) [36]. The white matter is more viscous than gray matter and responds less rapidly to mechanical loading that is imposed by the water hammer effect. In addition, the orientation of the major dendritic and axonal processes of gray matter are aligned with the vector of force delivered by the spinal fluid driven against the base of the sulcus, while the axonal processes of the U fibers in the white matter are oriented perpendicular to the vector of force. These two dynamics result in the shearing force and rupture of vessels near the interface of the U fibers with gray matter. Figure 5 illustrates this sequence. 

The authors recognize that the brain of patients with epilepsy also exhibit widening of the sulci [37] and the presence of biomarkers seen in CTE, such as phosphorylated tau [38]. Repeated impacts of the head against semi-rigid surfaces experienced during seizure episodes may lead to similar pathological processes in seizure disorders and CTE.

These observations are of interest because Kornguth and colleagues [39,40] had shown that CNS lesions in patients with neuropsychiatric systemic lupus erythematosus (NP SLE) had primary lesions in the same regions as observed by MRI. Antibodies against NF proteins were found in the NP SLE patient serum during exacerbations [39,40]. This observation suggests an increased vulnerability of the vascular matrix at the interface of white and gray matter to stress in general, including impact. 

### Proposed Mechanism for Development of CTE Following Repetitive Traumatic Events

**Figure 6:**
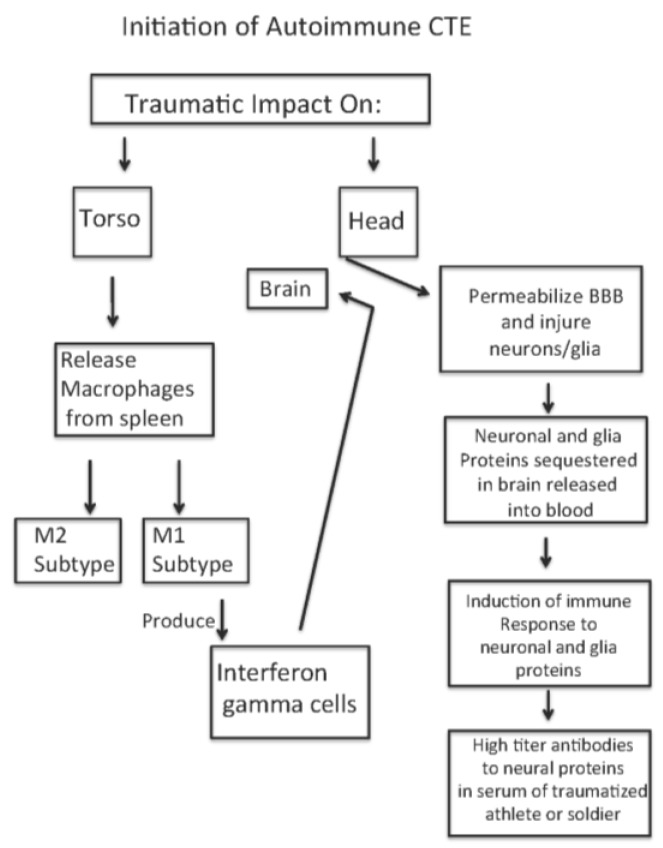
Sequence of Events Leading to Autoimmune Chronic Traumatic Encephalopathy (CTE) following Traumatic Impact on Athlete or Soldier. BBB: blood–brain barrier.

Our hypothesis is that high-force impact on the torso and head of soldiers exposed to IEDs or of athletes experiencing body impacts causes a release of macrophages from the spleen, an activation of the macrophages to M1 and M2 macrophage cells [20], and a transient increased permeability of the blood–brain barrier [41,42]. In addition, as a result of high-force impact of the head, neuronal and glial populations at the base of the sulci are injured and release proteins into the vascular compartment and CSF. These proteins generate antibody responses to the released proteins originally sequestered in the brain. With subsequent impact forces on the body, the activated M1 and M2 cells enter the brain parenchymal compartments across the permeablized BBB, as do the antineuronal and antiglial antibodies. The activated M1 cells produce interferon (IFN) gamma that then induces expression of MHC/HLA histocompatibility markers [43,44,45,46,47,48] by the neurons leading to neuronal silencing and subsequent death. Figure 6 illustrates this sequence of events. Support for this sequence of events is described below.

An excellent review of molecular, cellular, and system responses to traumatic head and body events is presented by Nizamutdinov and Shapiro [20]. There is a release of macrophages from the spleen into the blood which subsequently penetrates the CNS compartment at regions of disrupted BBB [41,42]. There are increases in levels of inflammatory response modifiers [46,47,48]. Finally, the entry of the activated macrophages through the permeabilized BBB will lead to the expression of elevated interferon gamma levels that then result in increased expression of MHC/HLA markers on the neurons [43,44,45,46,47], leading to silencing of the neuronal discharge and subsequent death.

The increased permeability of the BBB immediately following head impact and concussion will release neuronal and glial proteins (including neurofilament light, medium, and heavy [14,15,16,19]) from the CNS compartment. The released proteins may then initiate production of antibodies to the neuronal proteins, as shown by Kornguth in patients with small cell carcinoma of the lung [26,27]. During subsequent repetitive head injuries, the antibodies reactive/cross reactive to the neural proteins enter the CNS compartment and may cause the clinical signs of TBI. Each repetitive event is likely to exacerbate the injury, particularly when the interval between concussions is short. 

The increases in the l NF in serum are associated with initiation of anti-NF antibodies and indicative of an immune process as a major factor in the development of the clinical signs classified as TBI. The increased inflammatory response modifiers described in Nizamutdinov [20] are consistent with this hypothesis. In research from our laboratory, patients with small cell cancer of the lung (SCCL) that exhibited visual paraneoplastic syndrome were observed to have elevated levels of antibodies to the light chain neurofilament, and several of these patients had antibodies to the medium and heavy neurofilament proteins [26,27]. These antibodies to the NF proteins reacted with the small cell cancer as well as the large retinal ganglion cells affected by the visual paraneoplasia. The patients with these antibodies survived longer than those patients with SCCL alone. The anti-neurofilament antibodies resulted in the selective immunoablation of large retinal ganglion cells following injection of these antibodies into cat vitreous [28,29,30]. These observations on the SCCL population are supportive of the observation that the l NF proteins, seen in the mild to moderate TBI, pass from the neuronal compartment into the CSF over a prolonged time period. The continued release may serve as an antigenic stimulus to generate anti-NF antibodies in blood serum. This sequence of events whereby increased levels of l NF appears in the serum of patients at an early stage of progressive neurological disease is consistent with the finding of Byrne [49] that l NF appears to be an early biomarker correlated with the rate of clinical progression of the autosomal dominant disease Huntington Disease (HD). 

As described above, major research efforts funded by the National Institutes of Health, National Football League, the National Collegiate Athletic Association, and Defense Department have made efforts to detect biomarkers that are indicative of emerging CTE in persons exposed to high-impact forces as well as to define the pathological changes in the brain associated with CTE resulting from TBI [7,9,10,11,50]. These important investigations have identified neuronal and glial proteins that are released into CSF and blood compartments following impacts, and they also have identified increased amounts of tau and phosphorylated tau as well as neurofibrillary tangles in postmortem brains of athletes who had clinical CTE. The question that then arises is whether there is a specific protein that is responsible for both the initiation of the autoimmune process and for the clinical neurological sequalae that follow that are long term consequences of the process. The authors propose that following multiple traumatic impacts to the head and torso, different proteins are released and the immune response to these proteins is dependent upon the host organism. The factors controlling the variation include (1) activation of splenic macrophages to M1 and M2 subtypes; (2) levels of antibodies generated to the neuronal proteins present in serum and CSF; (3) changes in permeability of the BBB following impacts, fevers, toxicants that will facilitate entry of the macrophages and antibodies into the CNS compartments; (4) production of interferon gamma in the CNS; and, finally, the (5) rate of expression of MHC/HLA markers on the neuronal surface that leads to neuronal silencing and degeneration. This process, rather than singular events, is proposed by us to be the basis of variability among athletes and soldiers to head injury in the susceptibility to and development of the clinical manifestations called CTE. The observations of Bernick and colleagues [16]—that light NF increases in serum shortly after impact but then declines even while tau protein remains elevated—may suggest that the antibodies in serum that were produced following initiation of the autoimmune process bind the released light NF and thereby lead to a potential erroneous conclusion that there is no prolonged immune response to the NF antigen. The authors suggest that the subsequent impacts continue to release NF proteins that then further stimulate antibody production. The iterative process of antigen release and antibody boost together with the permeabilization of the BBB are proposed by us to be driving factors in the development of CTE. 

The hypothesis presented above indicates there is a likely association between traumatic head and torso impact injuries, the water hammer effect on the base of the sulci, resultant release of neurofilament protein, and generation of high titer antibodies to neurofilament protein that then leads to inflammatory responses, interferon gamma production by the monocytes in the CNS, and increased production of MHC markers on neurons. This sequence leads to neuronal silencing and the eventual development of CTE. To demonstrate that this autoimmune process is etiologically involved in developing CTE, the authors propose that, as the clinical symptoms of CTE begin to appear in the second and later decades following TBI, there may be marked increases in the titer of antineuronal/antiglial antibodies in spinal fluid and serum from the athletes or soldiers. If such correlations are made, strong evidence would be provided regarding an etiological role of the autoimmune process. 

As further evidence accumulates supportive of the hypothesis that autoimmune mechanisms are primary etiological events leading to CTE after traumatic brain injury, the strategies for mitigation include several approaches: treatment with statins [51,52], treatment with minocycline [53,54,55], antibodies to IFN gamma [56]. Statin treatment has been demonstrated to reduce the extent of brain damage following injury from stroke events [57]. The statins reduce the release of macrophages from the spleen, stabilize the blood–brain barrier, and decrease production of IFN gamma by the activated macrophages. The minocycline has use as an inhibitor of matrix metallo-proteinases where the proteinases disrupt the BBB [58]. Administration of minocycline in fact limits infarct size in humans by stabilizing the BBB [55]. Kantarci and colleagues [59] have discussed the possible use of anti-interferon gamma antibodies in the treatment of patients with autoimmune diseases. This treatment is also suggested by the study of Zhao [60], where it is demonstrated that interferon gamma is expressed at higher levels in pathogenic Th 17 cells compared with cholera-toxin-induced Th 17 cells. However, recent studies [61] suggest that interferon gamma may have both positive and negative effects on the progression of autoimmune diseases depending upon the length of illness, sex of patient, and other factors. The statins and minocycline may offer more promising avenues of exploration than the anti-interferon gamma treatment. 

Additionally, the observations of Dretsch and colleagues [62] that individuals with brain-derived neurotropic factor with Val 66 Met modification are significantly more likely to develop CTE following impact injuries provides a capability for screening of individuals prior to exposure. Pretesting of soldiers and athletes for this brain-derived neurotrophic factor (BDNF) form could markedly reduce exposure and hence long-term risk of the individual for CTE. 

## 5. Conclusions

From our study of university-level women soccer athletes followed over a period of four and a half years the authors report several significant findings: first, a marked increase in the width of sulci in the frontal to occipital cortices; second, an appearance of subtle hemorrhagic changes at the base of the sulci; third was a sustained reduction in total brain volume in several soccer players at a developmental time when brain growth is generally seen. The changes documented by MRI represent a clue to the pathological mechanism following an injury paradigm. The authors propose a mechanism of injury in CTE that is caused by an autoimmune process associated with the release of neural proteins from nerve cells at the base of the sulcus from a water hammer injury effect. As evidence accumulates to support this hypothesis, there are pharmacological treatment strategies that may be able to mitigate the development of long-term disability from TBI.

## Figures and Tables

**Table 1:** IMPACT (Immediate Post-Concussion Assessment and Cognitive Testing) data on the soccer athletes studied. The rows with no asterisk are baseline measures prior to concussive events received during collegiate competition. The rows with one asterisk are data acquired within 48 h after concussion. The row with a single asterisk for Players 15 and 29 includes data obtained within 48 h after concussion. The row with two asterisks for Player 15 includes data acquired 5 days after concussion.

Player	Memory Verbal	Memory Visual	Motor	Reaction Time	Impulse Control	Total Symptoms	Cognitive Efficiency
10	80	83	48	0.47	8	10	0.28
11	100	88	51	0.6	7	3	0.37
12	88	77	38	0.54	14	5	0.37
13	85	58	33	0.72	1	3	0.37
14	85	70	43	0.68	3	28	0.24
15	77	73	42	0.54	7	6	0.2
15 *	62	73	22	1.17	2	18	0.08
15 **	88	83	41	0.6	7	1	0.49
16	100	85	37	0.56	2	5	0.35
17	100	94	42	0.64	3	1	0.39
29	85	57	41	0.5	6	6	0.47
29 *	88	74	38	0.49	6	0	0.37
30	100	80	46	0.7	7	13	0.02
